# Adaptation and Validation of the Serbian Version of Dyslexia Screening Test-Junior

**DOI:** 10.3390/children12030322

**Published:** 2025-03-02

**Authors:** Tanja Lukovac, Vitomir Jovanović, Svetlana Petronijević, Jovana Radović, Neda Milošević Dedakin, Tatjana Savić, Dragan Pavlović

**Affiliations:** 1 Center for Speech and Language Pathology Higia Logos, 11000 Belgrade, Serbia; 2 Faculty of Philosophy, University of Belgrade, 11000 Belgrade, Serbia; vitomirj@gmail.com; 3 Faculty of Sport, University “Union—Nikola Tesla”, 11000 Belgrade, Serbia; 4 Department of Speech and Language Pathology, College of Human Development, 11000 Belgrade, Serbia; 5 Institute for Biological Research “Siniša Stanković”—National Institute of the Republic of Serbia, University of Belgrade, 142 Despot Stefan Boulevard, 11000 Belgrade, Serbia; tanjat@ibiss.bg.ac.rs; 6 Faculty of Special Education and Rehabilitation, University of Belgrade, 11000 Belgrade, Serbia

**Keywords:** DST-J, dyslexia, Serbian language, primary school children

## Abstract

**Background/Objectives**: Dyslexia, a prevalent reading disorder, substantially affects children’s educational and social development. Early diagnosis is essential for timely intervention; however, Serbia lacks a standardized instrument for assessing dyslexia in primary school children. This study aimed to evaluate the reliability and predictive validity of the Dyslexia Screening Test-Junior (DST-J), which was adapted for the Serbian language and cultural context. **Methods**: The study sample comprised 647 children from the general population, aged 6 years and 6 months to 11 years and 5 months, along with 30 children of comparable age exhibiting reading difficulties. The assessment of validity was based on the at-risk quotient, predictive validity, and test–retest reliability. **Results**: Significant differences in at-risk quotient (ARQ) scores were found between children with and without dyslexia (t = 14.90, *p* < 0.001), with boys, particularly those aged 9.6 to 10.5 years, having higher risk scores than girls. Internal consistency was acceptable (Cronbach’s α = 0.704), and construct validity was confirmed by correlations with external measures, which explained 44% of the variance (R^2^ = 0.44; *p* < 0.01). Predictive validity was high for key subtests such as rapid naming and phonemic segmentation, reaching maximum accuracy (sensitivity and specificity = 1). **Conclusions**: The findings indicate that the adapted DST-J is effective in identifying dyslexia risk among Serbian primary school children. The innovation of this study lies in the cultural adaptation of the DST-J, with future research directed towards refining this instrument and exploring additional diagnostic criteria to enhance its accuracy and inclusivity.

## 1. Introduction

Dyslexia is the most common reading disability, often characterized by persistent difficulties with reading accuracy, spelling, and phonological awareness. Children with dyslexia often struggle with problems such as reduced reading fluency, slow and effortful reading, frequent errors, orthographic errors, fatigue when reading or writing, difficulty organizing thoughts on paper, and impaired verbal memory [1]. Deficits in phonological awareness are considered the hallmark of dyslexia.

Advances in neuroimaging techniques, such as functional magnetic resonance imaging, have revealed a clear neurobiological signature of dyslexia [2]. Neuropsychological studies also suggest that the primary mechanism underlying learning difficulties in dyslexia is a deficit in phonological processing [3]. Despite these findings, the exact etiology of dyslexia remains unclear as it results from several interacting factors. These include deficits in linguistic encoding [4], impaired processing of rapid temporal stimuli [5], and abnormal functioning of the neural nodes responsible for integrating information across cognitive systems [6]. Dyslexia also has a strong genetic basis, with estimates of heritability ranging from 40% to 80% [7]. Studies consistently report a higher prevalence of dyslexia in boys, who are two to three times more likely to be affected than girls [8].

Although dyslexia is universal, its prevalence and methods of diagnosis vary from language to language [2]. Dyslexia affects 5 to 10% of primary school children worldwide [9]. In languages with transparent orthography, such as Serbian, the prevalence is generally lower; for example, only 3% of Italian children are affected [10]. Currently, there is no reliable data on the prevalence of dyslexia in Serbia, mainly due to the lack of a standardized diagnostic tool tailored to the Serbian language and cultural context. As there are no precise definitions and standardized criteria for the diagnosis of dyslexia, adapting test batteries to new linguistic and cultural circumstances is a major challenge [11], as it can lead to unexpected variations in the relationships between subtests, such as the anticipated stronger or weaker associations between specific subtests involved in reading acquisition. In Serbia, however, there is an urgent need for a valid, comprehensive diagnostic method, as no existing tool adequately assesses all risk factors for dyslexia in primary school children.

The Dyslexia Screening Test-Junior (DST-J test) can be used to identify children at risk of dyslexia. It was introduced in England in 1996 [12] and is intended for children aged 6 years and 6 months to 11 years and 5 months [13]. The DST-J provides a direct assessment of participants’ reading and spelling abilities and assesses various skills that may indicate a risk of dyslexia [13]. This test, originally developed for English and later translated and adapted into Dutch (DST-NL) [14], has been shown to be effective in assessing technical skills, but is not a perfect instrument for the comprehensive diagnosis of dyslexia, particularly in children living in a multicultural environment such as the Netherlands [15]. The DST-J was also developed in Malaysia, where the main challenge was to accurately define dyslexia and select appropriate parameters based on existing theories and definitions to develop an accurate and valid test battery for the Malay language [16]. The DST-J and similar dyslexia screening test variants are available in both web-based and online formats [17].

The DST-J requires linguistic and cultural adaptation, as it was originally developed for the English language, as well as normative data to accommodate the local population. The test items (phonological awareness, memory, automaticity) may also need to be adapted to the linguistic characteristics and educational systems of different countries in order to be validly applied [18]. Since DST-J was created for the English language, which is a non-transparent and alphabetic language, an adaptation to the Serbian language, which is transparent and uses the Cyrillic alphabet, is required.

The aim of this study was to assess the validity of the adapted Dyslexia Screening Test-Junior (DST-J) as a screening tool for dyslexia in Serbian primary school children. The DST-J consists of 12 subtests that include performance measures (reading, writing, and spelling) as well as diagnostic items to identify the causes of learning difficulties. By generating a cumulative at-risk quotient (ARQ), the DST-J provides an initial assessment that, in combination with clinical assessment, allows for early intervention. This work aims to fill the diagnostic gap in Serbia by providing a reliable tool for identifying children at risk of dyslexia and supporting their educational and social development.

## 2. Materials and Methods

**Participants.** This study involved one experimental group that comprised 647 children (47.9% girls) from five primary schools in Belgrade, ranging from 1st to 5th grade (ages 6 years and 6 months to 11 years and 5 months). The distribution of boys and girls by age groups is given in Table 1. Inclusion criteria encompassed normal intellectual functioning. Exclusion criteria included bilingualism, evident somatic, neurological, psychiatric, and sensory disorders, and below-average intellectual levels. A comparative analysis was conducted using a positive (boys with dyslexia, n = 30) and a negative (boys without dyslexia, n = 32) control group. The children of positive and negative control are of the same age between 9 and 10 years old. Prior to the study, the negative control group was thoroughly tested to ensure that none of the participants exhibited dyslexic traits by direct assessments of reading and spelling skills. The participants in the positive control were diagnosed with dyslexia at the Department of Psychophysiological Disorders and Speech Pathology “Prof. Dr Cvetko Brajović” in Belgrade. The control groups were carefully matched on key variables, including age, school, sex, and class, to account for potential confounding factors.

**DTS-J test battery in Serbian language.** The adaptation and validation of the DST-J in Serbian was based on the manual [18] and the guidelines of the International Test Commission [19]. When adapting the DST-J test battery to the Serbian language, the original 12 subtests were retained: rapid naming (the ability to name words or objects quickly and automatically), bead stringing (how many beads the child could string in a given amount of time), one-minute reading (the number of words the child could read correctly in a given amount of time), postural stability (measuring balance difficulties), phonemic segmentation (12 words are read to the child one at a time and after each word the child is asked to segment the word by crossing out a syllable, consonant or vowel), two-minute spelling (how many words a child can write in the given amount of time), nonsense passage reading (to assess the child’s ability to read unfamiliar words), backward digit span (a child is asked to repeat numbers backwards), one-minute writing (a target to see how quickly and accurately a child can copy the given text), verbal fluency (to assess cognitive function), semantic fluency (to correctly name as many words as possible from a single category in a given amount of time), and vocabulary (16 words are read to the child one at a time and after each word the child is asked to circle the picture on the paper). The results of these subtests are combined to produce an ARQ of dyslexia. DST-J test battery was translated into Serbian and culturally adapted following standard guidelines [20].

**Testing conditions.** Given the test’s high sensitivity, specific conditions were required: a quiet, well-lit room equipped with tables and chairs, and each table containing the necessary materials, including a balance tester, beads and string, a stopwatch, and score sheets. All children were assessed using the DST-J testing battery. Each testing session lasted approximately 25 min per child. A retest was conducted four months later on a sample of 64 randomly selected children from the control group under identical conditions. Data collection spanned seven months.

**Measures and Scoring.** All children were tested with the adapted versions of the DST-J subtests and all 12 of these are included in the final score (ARQ). Firstly, a comparative analysis of the ARQ for dyslexia was conducted to evaluate the performance of participants in both the positive and the negative control groups. Furthermore, the validity of the adapted DST-J was evaluated using the Wechsler Intelligence Scale for Children—Revised (WISC-R; [21]), administered to a group of 40 randomly selected children 15 days after the retest. Finally, a profile sheet was drawn up for each child and the overall results of the individual tests were collated. The results were compared to the DST-J scoring manual guidelines to determine the severity of dyslexia. These comparisons helped to determine the appropriate categorization of each child’s performance. The scores obtained were then used to calculate the ARQ for each child by adding the results of the subtests and dividing by 11. An ARQ greater than 1.2 indicated a high risk of dyslexia, a quotient between 0.9 and 1.2 indicated a medium risk, and a quotient between 0.6 and 0.9 indicated a low risk. The one-minute reading subtest from the DST-J and the vocabulary subtest from the WISC-R served as validity criteria to explore the relationship between vocabulary deficits and ARQ scores. Students’ grades in Serbian language, vocabulary development (from WISC-R), and teachers’ assessments of reading quality were used as additional measures for construct validity. Grades were obtained at the end of the assessment period as final grades, while reading ability was described qualitatively by teachers. These descriptions were rated on a numerical scale from one to five. For the negative control, teachers were instructed to exclude children with any reading difficulties. The positive control group consisted of students diagnosed with Mixed Disorder of Scholastic Skills (F81.3), with a predominance of reading disorders, at the Department of Psychophysiological Disorders and Speech Pathology ’Prof. Dr. Cvetko Brajović’ in Belgrade.

**Statistical Analysis.** Statistical analyses were conducted using SPSS (version 20.0.0, IBM, Armonk, NY, USA) and STATISTICA (version 8.0, StatSoft, Tulsa, OK, USA), while Confirmatory Factor Analysis (CFA) were performed in Phyton (3.10.1).

The validity of the DST-J battery on the Serbian language was assessed by confirmatory factor analysis (CFA). After confirming the measurement model, a structural equation modeling (SEM) analysis was conducted to examine the causal relationships between the factors. The model was assessed using the diagonal weighted least squares (DWLS) method. Model fit was assessed using the relative chi-squared index (χ^2^/df), comparative fit index (CFI), Tucker–Lewis index (TLI), root mean square error of approximation (RMSEA), and standardized root mean square residual (SRMR). Fitting was considered appropriate when CFI and TLI ≥ 0.90–0.95, RMSEA ≤ 0.08–0.06, and SRMR ≤ 0.10–0.08 [22]. A multifactorial model comprising three latent factors was tested. The model is defined by different observed variables that serve as indicators for each latent construct. Each factor is represented by the following observed indicators: Factor 1 (F1)—Reading and writing skills (one-minute reading score, one-minute writing score, nonsense text reading score, two-minute spelling score, and stringing beads score); Factor 2 (F2)—Working memory (backward number sequence score, vocabulary score, verbal fluency score, postural stability score, and semantic fluency score); Factor 3 (F3)—Phonological awareness (rapid naming score and phonemic segmentation score).

Data normality was assessed using the Kolmogorov–Smirnov test. To stabilize variance, ARQ values were square-root transformed. A t-test was used to evaluate the ARQ score of the DST-J test between positive and negative control groups. A two-way analysis of variance (ANOVA) was performed to examine the effects of gender and age on ARQ, followed by a post hoc test for least significant differences where applicable. Spearman correlations were utilized to evaluate the relationship strength between test and retest scores of the subtests, and Cronbach’s alpha was computed to estimate the internal consistency of the DST-J subtests. Construct validity of the DST-J battery was analyzed using linear regression and standardized beta coefficients to compare the DST-J and WISC-R tests. Predictive validity was estimated using the “caret” and “caTools” libraries in the R programming language. Data were divided into training and test sets in a 0.7:0.3 ratio (441 subjects, including 21 with dyslexia, in the training set and 189 subjects, including 9 with dyslexia, in the test set).

## 3. Results

### 3.1. CFA of the DST-J Test Validity in Serbian Language

CFA applied to the Serbian-translated version of the DST-J demonstrated an acceptable fit for the original three-factor model. The model fits the data well (χ^2^(51) = 138.99, *p* < 0.001), and given the sample size, additional indices confirm its adequacy. CFI (0.987) and TLI (0.983) indicate an excellent fit, while RMSEA (0.052) and SRMR (0.048) suggest minimal deviations, confirming that the model accurately represents the data structure. Detailed statistical parameters are provided in the Supplementary Materials.

All indicators exhibited significant and high factor loadings (*p* < 0.05), supporting the model’s strong convergent validity. Factorial analysis of variance and covariance revealed statistically significant differences in the strength of latent factors, as well as differences between them. F1 showed the highest variance (λ = 347.28), followed by F3 (λ = 90.76), while F2 had the lowest (λ = 0.277), with stable confidence intervals. Covariances between F1 and F2 were positive (λ = 8.049), whereas those between F1 and F3 (λ = −157.027) and F2 and F3 (λ = −5.043) were negative, indicating distinct dimensions among the factors.

Based on the results of the performed CFA, we can conclude that the Serbian version of the DST-J test maintains the original factorial structure of the DST-J, exhibiting a multidimensional framework consistent with the original model.

### 3.2. Discriminative Power and ARQ Score Analysis

A comparative analysis confirmed that the adapted DST-J effectively differentiates between children with and without dyslexia. Boys diagnosed with dyslexia (n = 30) had significantly higher ARQ scores compared to boys without dyslexia (n = 32). The t-test revealed a highly significant difference (t = 14.90, *p* < 0.001) with a very large effect size (Cohen’s d = 4.71), indicating a considerable performance gap. This result emphasizes the ability of the DST-J to discriminate children at risk of dyslexia in the Serbian population and supports its use as a reliable screening tool.

The ARQ scores of 647 children were further analyzed to examine age and gender differences. Table 2 shows the mean ARQ scores by age and gender, and Table 3 summarizes the distribution of children across the four risk levels of dyslexia. For boys, mean ARQ scores increased with age, peaking in the 9.6 to 10.5 age group. Conversely, ARQ scores in girls decreased with age, with the highest scores observed in the youngest group (6.6 to 7.5 years). A two-way analysis of variance (ANOVA) revealed that boys had significantly higher ARQ scores than girls (F = 19.76, df = 1; *p* < 0.001), and ARQ scores differed significantly between age groups (F = 5.007, df = 4; *p* < 0.001). The post hoc analysis showed that children aged 7.6 to 8.5 years had significantly lower ARQ scores than older children (*p* < 0.001). The interaction between gender and age was also significant (F = 2.912, df = 4; *p* < 0.05), which shows that the influence of age on ARQ scores varies depending on gender. Regarding dyslexia risk, 26.72% of boys in the oldest age group (9.6 to 10.5 years) were categorized as high risk, while the highest percentage of girls at high risk (11.94%) was found in the youngest age group (6.6 to 7.5 years). Younger boys and older girls were more often categorized as low risk. These results show the importance of considering both age and gender when interpreting DST-J results.

### 3.3. Internal Consistency of the Subtests

Descriptive statistics of scores on subtests in the Serbian version of DST-J test are given in Table 4. Distribution of scores for bead threading, one-minute reading, backwards digit span and semantic fluency are approximately symmetric, platykurtic and with flat peak. One-minute reading is characterized by a pronounced flattened distribution suggesting that the data are more spread out around the mean. Values for score in subtests rapid naming, postural stability, two-minute spelling, and verbal fluency are small, highly right-skewed, leptokurtic (the data are highly concentrated around the mean), and with a sharp peak. Highly left-skewed data with platykurtic distribution and a flat peak is observed in the phonemic segmentation and nonsense passage reading subtests. Also, platykurtic distribution and flat peak was observed in the vocabulary (moderately left-skewed data) and one-minute writing (moderately skewed data) subtests. The reliability analysis was carried out for the perceived ARQ for 12 subtests. Cronbach’s alpha showed that the tests achieve acceptable reliability (Cronbach’s α = 0.704). Most of the subtests are worth retaining, as their deletion led to a reduction in alpha (Table 5). The only exception was the vocabulary subtest, where Cronbach’s alpha increased slightly to 0.705. Therefore, the deletion of this subtest should be considered.

### 3.4. Construct Validity of the DST-J Test Battery

The construct validity of the DST-J test battery was confirmed, although the external measures can be influenced by factors other than dyslexia and the sample used to test construct validity is very small (N = 40). The predictors of dyslexia risk were grades in Serbian language, vocabulary development (according to the WISC-R test categories) and the teacher’s assessment of reading quality. The linear regression model with the three predictor variables mentioned explains about 44% of the variance (R2 = 0.44; *p* < 0.01), which, given the small number of subjects, represents results that show satisfactory construct validity. The best predictor of dyslexia risk measured with the DST-J test battery is the WISC-R vocabulary score. As expected, all predictors are negatively correlated with the DST-J score (Table 6).

### 3.5. Predictive Validity of the DST-J Test Battery

Predictive validity analysis was used to test the probability of the presence of dyslexia as a function of age and the total score of the DST-J test battery and to confirm the accuracy of the test. The results showed that the DST-J test can be used as a valid instrument to serve as a predictive indicator for further formal diagnostic tests. The results indicate that the predictive validity is real and not a statistical artifact. The predictive logistic regression model took into account the standardized score of the entire DST-J test battery and the age of the test subjects as predictors. The estimated parameters indicated the maximum possible validity of the test (accuracy = 100%, area under the curve = 1, kappa = 1, specificity = 1, sensitivity = 1). All boys with a dyslexia diagnosis (9 out of 9) in the test dataset were correctly classified, with no false positives or false negatives, i.e., with maximum recall and sensitivity of the test. The results showed that a change of one standard deviation in the rapid naming subtest increased the probability of dyslexia in a child by 7.45 times. A change of one standard deviation in the one-minute reading and fluency subtests increased the likelihood of dyslexia by 6.7 and 6 times, respectively. Subtests with higher predictive value included reading nonsense passages, two-minute spelling, phonetic segmentation, and one-minute writing. The subtests for backward digit span, semantic fluency, vocabulary, and bead threading showed low predictive value (Table 7) and have a test–retest reliability of <0.70 (Table 7 and Table 8). The differences associated with the different age groups indicate the need for standardization for the age groups, although there was some overlap for the different age groups. Postural stability was not a statistically significant predictor. Sub-tests that are statistically significant (*p* < 0.01) contribute to the identification of children with dyslexia.

### 3.6. Test–Retest Reliability

The Spearman correlation between test and retest of the individual subtests of the DST-J test shows that all subtests correlate positively. Most of them show significant test–retest reliability (Table 8). Only the fluency subtest had moderate test–retest reliability, and the postural stability subtest had low test–retest reliability. Within the DST-J test battery, the retests for the rapid naming, one-minute reading, phonemic segmentation, two-minute spelling, reading nonsense passages, and one-minute writing subtests had a common variance ranging from 52% to 93%. The repetition tests for bead threading, postural stability, backward digit span, verbal fluency, semantic fluency, and vocabulary subtests had a common variance in the range of 6% to 48%.

## 4. Discussion

Dyslexia is one of the most common learning disabilities, but its identification and definition varies from language to language [2,11]. The aim of this study was to validate the DST-J testing battery for use in the Serbian language and cultural context and thus close the gap in standardized diagnostic instruments for Serbian primary school children. Early detection of dyslexia is crucial for improving academic performance, social integration, and general quality of life, as well as for reducing social and family stress. The DST-J was selected for adaptation due to its comprehensive design, which assesses multiple domains related to reading impairment. The primary objective was to evaluate the predictive validity and reliability of the DST-J in identifying dyslexia risk in Serbian children. Our results confirm that the DST-J can serve as a reliable and valid instrument for dyslexia screening in Serbia and contribute to early intervention.

An important finding of this study is the effect of gender and age on the ARQ scores. Boys, especially older ones, showed higher ARQ scores compared to girls, which is consistent with previous research findings indicating that males often have greater reading difficulties [23]. In contrast, girls’ ARQ scores decreased with age, with the highest ARQ scores observed in younger girls. These gender differences in dyslexia risk are consistent with global findings suggesting that boys are at higher risk for reading difficulties [2,11]. There is a strong reduction in the risk of dyslexia in girls as early as the second age group.

The internal consistency of the subtests within the adapted DST-J showed acceptable reliability (Cronbach’s α = 0.704). The deletion of one subtest led to a reduction in reliability, indicating that all subtests made a meaningful contribution to the overall score. The exception was the vocabulary subtest, where deletion slightly increased the Cronbach’s alpha to 0.705, indicating that it is not essential for predictive accuracy. Test–retest reliability was generally high, with significant correlations for most subtests, confirming the stability of the DST-J over time. However, some subtests, such as bead threading, postural stability, backward digit span, verbal fluency, and semantic fluency, showed lower test–retest reliability and a common variance of 6% to 48%. These results suggest that although these subtests contribute to the overall test, their individual predictive value may be limited.

In our study, factor analysis showed that Factor 2 has a lower factor loading (λ = 0.277), but this is probably due to the use of unstandardized subtest scores, which preserves the interpretability of variances and loadings in their natural units. This does not indicate inadequate assessment of this factor in the Serbian version. This approach enables direct comparison of factor variances, ensuring that results accurately reflect the observed measures. To further confirm the robustness of our findings, we conducted an additional analysis using unstandardized estimates (see Table S5 in the Supplementary Materials). These findings align with research on Baddeley’s working memory model [24], highlighting individual differences in working memory capacity as key to knowledge acquisition, skill development, and reading comprehension [25,26]. While verbal short-term memory aids letter recognition, its role is secondary to phonological awareness, which plays a more critical role in reading development [27,28]. These results underscore the complexity of cognitive processes, demonstrating that their roles in learning and language cannot be reduced to single factors.

Construct validity was confirmed by examining the relationship between the one-minute reading subtest of the DST-J and the vocabulary subtest of the WISC-R and showed a strong correlation. This suggests that reading performance as measured by the DST-J is a reliable indicator of dyslexia risk, which is also confirmed by external measures such as language grades and teacher ratings [29]. These findings are consistent with previous research emphasizing the role of oral language skills in reading achievement [30,31]. Many authors suggest that a well-developed vocabulary improves reading efficiency and enables faster comprehension of text [32,33,34]. The predictive validity of the DST-J was particularly high for the subtests on reading nonsense passages, two-minute spelling, phonetic segmentation and one-minute writing. A change of one standard deviation in these subtests significantly increased the likelihood of dyslexia and confirmed their usefulness in identifying at-risk children. Phonological awareness, an important predictor of reading difficulties, is particularly important in transparent orthographies such as Serbian, as phonetic consistency increases the reliability of such measures [35,36,37]. The high predictive validity of phonetic segmentation emphasizes its importance in dyslexia screening in the Serbian context [38,39].

The predictive value of the verbal and semantic fluency subtests emphasizes the role of phonological and semantic representations in word recognition and supports the theory that children with dyslexia have deficits in these areas [40,41]. These findings are consistent with previous research showing that phonological skills are crucial for reading development, especially in transparent orthographies such as Serbian [42,43].

Despite the overall high predictive validity of the DST-J, certain subtests, including backward digit span, postural stability and vocabulary, showed low predictive value and low test–retest reliability. The backward digit span, which is commonly used in IQ tests, showed low performance scores in dyslexic children, which is confirmed in the literature [44]. In addition, the postural stability subtest showed limited reliability, raising questions about its effectiveness in screening for dyslexia [45]. This is consistent with findings that balance difficulties in dyslexic children may not be a consistent predictor when attention is not diverted from the task [44].

Limitations and Future Directions: Further studies, including assessments with diverse participant populations, are needed to fully validate the Serbian version of the DST-J test. A limitation of our study is the lower variance observed for F2 (working memory), likely due to the use of unstandardized subtest scores to preserve factor interpretability. Although previous research anticipated higher variance for F2, the use of unstandardized subtest scores may have contributed to the lower value observed. Future research could explore the effects of standardizing subtest scores to better assess the variance in working memory. WISC-R was chosen for construct validity due to the limited availability of validated instruments in Serbian. Despite this limitation, the predictive validity results support the instrument’s effectiveness in identifying children with dyslexia. Future research should focus on validating the DST-J with additional instruments and statistical analyses, as well as refining the test through further psychometric evaluation.

## 5. Conclusions

In this study, the DST-J test battery was successfully adapted and validated for use with Serbian primary school children. The results show that the DST-J is a reliable and valid instrument for the early detection of dyslexia in Serbia. The main results showed that ARQ scores varied significantly with age and gender, with boys, especially older children, being at higher risk of dyslexia than girls. The internal consistency of the subtests was acceptable, and the test–retest reliability confirmed the stability of the DST-J over time. Construct validity was supported by significant correlations between the one-minute reading subtest of the DST-J and the vocabulary subtest of the WISC-R. The DST-J also showed high predictive validity for key subtests such as reading nonsense passages, two-minute spelling and phonetic segmentation, making it an effective tool for identifying children at risk of dyslexia. While some subtests, such as backward digit span and postural stability, had lower predictive value, the DST-J battery proved to be a robust screening tool overall. In summary, this study represents a crucial step towards addressing the lack of standardized instruments for the assessment of dyslexia in Serbia. The adapted DST-J can be used by educators and clinicians to identify dyslexia risks at an early stage, enabling timely intervention and support for affected children and thus improving their academic and social outcomes. Further refinement of certain subtests and broader standardization efforts across age groups will increase the utility of this tool.

## Figures and Tables

**Table 1:** Distribution (%) of boys and girls by age groups.

	Gender		
Age Group (Years)	Boys	Girls	Total
6:6–7:5	10.089	10.645	10.355
7:6–8:5	21.068	26.129	23.493
8:6–9:5	22.255	21.935	22.102
9:6–10:5	20.475	15.161	17.929
10:6–11:5	26.113	26.129	26.121

**Table 2:** At-risk quotient values.

Age Group (Years)	Gender (Mean ± SE)		Total (Mean ± SE ^1^)
	Boys	Girls	
6:6–7:5	0.73 ± 0.09	0.82 ± 0.09	0.80 ± 0.06
7:6–8:5	0.74 ± 0.06	0.58 ± 0.05	0.65 ± 0.04
8:6–9:5	0.86 ± 0.05	0.72 ± 0.05	0.79 ± 0.04
9:6–10:5	1.02 ± 0.05	0.66 ± 0.05	0.87 ± 0.04
10:6–11:5	0.95 ± 0.05	0.67 ± 0.04	0.82 ± 0.03

^1^ SE = standard error.

**Table 3:** Distribution of children of different ages for four-levels of the at-risk quotient.

Risk	Gender	Age Group (Years)				
		6:6–7:5	7:6–8:5	8:6–9:5	9:6–10:5	10:6–11:5
No risk	Boys	22.39	20.39	14.69	8.62	12.43
	Girls	14.93	31.58	19.58	16.38	21.30
	Total	37.31	51.97	34.27	25.00	33.73
Low risk	Boys	13.43	13.82	16.08	15.52	11.24
	Girls	13.43	9.21	16.78	14.66	15.38
	Total	26.87	23.03	32.87	30.17	26.63
Mild risk	Boys	5.97	4.61	10.49	8.62	11.24
	Girls	8.96	6.58	6.29	6.03	6.51
	Total	14.93	11.18	16.78	14.66	17.75
High risk	Boys	8.96	7.89	11.19	26.72	17.16
	Girls	11.94	5.92	4.90	3.45	4.73
	Total	20.90	13.82	16.08	30.17	21.89

**Table 4:** Descriptive statistics of scores on subtests in the Serbian version of DST-J test.

Subtests	Mean ± SD	Skewness	Kurtosis
Rapid naming	48.014 ± 18.227	3.271	17.027
Bead threading	5.317 ± 1.718	0.171	0.792
One-minute reading	46.216 ± 22.303	0.349	−0.547
Postural stability	2.716 ± 2.934	1.559	5.217
Phonemic segmentation	8.470 ± 2.786	−1.159	0.818
Two-minute spelling	9.739 ± 3.903	1.293	2.713
Backwards digit span	2.433 ± 1.034	0.181	0.847
Nonsense passage reading	53.250 ± 18.606	−1.486	1.627
One-minute writing	7.889 ± 4.063	0.743	0.721
Verbal fluency	8.893 ± 3.932	2.313	20.008
Semantic fluency	16.056 ± 5.233	0.360	0.500
Vocabulary	13.085 ± 1.535	−0.661	1.154

**Table 5:** Internal consistency of DST-J test using Cronbach’s α values—omitted item statistics; list of subtests whose exclusion results in a decrease in Cronbach’s α values.

Subtest	Cronbach’s Alpha
Rapid naming	0.680
Bead threading	0.701
One-minute reading	0.664
Postural stability	0.702
Phonemic segmentation	0.662
Two-minute spelling	0.681
Backwards digit span	0.686
Nonsense passage reading	0.667
One-minute writing	0.693
Verbal fluency	0.683
Semantic fluency	0.693
Vocabulary	0.705

**Table 6:** DST-J score prediction according to external measures.

		Correlation Coefficient (r)		
	β Coefficient	Original	Partial	Semi-Partial
WISC—R test	−1.013	−0.573	−0.297	−0.233
Serbian language grade	−0.211	−0.573	−0.151	−0.114
Reading description	−0.332	−0.538	−0.242	−0.186

**Table 7:** Standardized scores on subtests as predictors.

Standardized Scores on Subtests	Odds Ratio (Exp(β))	*p*-Value
Rapid naming	7.45	<0.01
One-minute reading	6.69	<0.01
Verbal fluency	6.00	<0.01
Nonsense passage reading	5.50	<0.01
Two-minute spelling	5.40	<0.01
Phonemic segmentation	5.30	<0.01
One-minute writing	3.60	<0.01
Backwards digit span	1.10	>0.05
Age	1.10	>0.05
Semantic fluency	1.08	>0.05
Vocabulary	1.01	>0.05
Bead threading	1.01	>0.05

**Table 8:** Spearman coefficient (*r*) and significance of test–retest reliability.

Subtest	Spearman Coefficient (*r*)					
	Boys	*p*-Value	Girls	*p*-Value	Both	*p*-Value
Rapid naming	0.813	<0.001	0.700	<0.001	0.768	<0.001
Bead threading	0.658	<0.001	0.647	<0.001	0.639	<0.001
One-minute reading	0.928	<0.001	0.976	<0.001	0.959	<0.001
Postural stability	0.346	<0.05	0.095	>0.05	0.244	>0.05
Phonemic segmentation	0.737	<0.001	0.749	<0.001	0.734	<0.001
Two-minute spelling	0.768	<0.001	0.867	<0.001	0.807	<0.001
Backwards digit span	0.689	<0.001	0.397	<0.05	0.571	<0.001
Nonsense passage reading	0.790	<0.001	0.909	<0.001	0.841	<0.001
One-minute writing	0.816	<0.001	0.769	<0.001	0.810	<0.001
Verbal fluency	0.284	>0.05	0.652	<0.001	0.472	<0.001
Semantic fluency	0.693	<0.001	0.622	<0.001	0.661	<0.001
Vocabulary	0.530	<0.001	0.529	<0.01	0.544	<0.001
At-risk quotient	0.702	<0.001	0.715	<0.001	0.704	<0.001

## Data Availability

The authors confirm that the data supporting the findings of this study are available within the article.

## Supplementary Materials

The following supporting information can be downloaded at: https://www.mdpi.com/article/10.3390/children12030322/s1, Table S1. Model fit indices and metrics for confirmatory factor analysis (T-size CFI is computed for α = 0.05. The T-size equivalents of the conventional CFI cut-off values (poor < 0.90 < fair < 0.95 < close) are poor < 0.868 < fair < 0.928 < close for model: Model 1; T-size RMSEA is computed for *α* = 0.05. The T-size equivalents of the conventional RMSEA cut-off values (close < 0.05 < fair < 0.08 < poor) are close < 0.06 < fair < 0.089 < poor for model: Model 1.) Table S2. Standardized factor loadings with confidence intervals (95% CI). (Estimate—estimated factor load; Std. Error—standard error; 95% CI—confidence interval of 95%); Table S3. Factor analysis, parameter estimates and 95% confidence intervals for latent variables (Estimate—estimated factor load; Std. Error—standard error; 95% CI—confidence interval of 95%) ; Table S4. Factor covariance estimates and 95% confidence intervals for latent variable pairs (Estimate—estimated factor load; Std. Error—standard error; 95% CI—confidence interval of 95%); Table S5. Residual variances and 95% confidence intervals for unstandardized variables (Estimate—estimated factor load; Std. Error—standard error; 95% CI—confidence interval of 95%).

## Abbreviations

The following abbreviations are used in this manuscript:

ARQ	At-Risk Quotient
DST-J	Dyslexia Screening Test-Junior
