# Supplementary material for: Adaptation and Validation of the Serbian Version of Dyslexia Screening Test-Junior
Source: Children (Basel). 2025 Mar 2;12(3):322. doi: 10.3390/children12030322 (PMC11941116; doi:10.3390/children12030322)
Supplement: Supplementary file 1 [file children-12-00322-s001.zip › children-3455056-supplementary.pdf]

Supplementary Material

Confirmatory Factor Analysis (CFA)

Table S1. Model fit indices and metrics for confirmatory factor analysis (T-size CFI is computed for $\alpha = 0.05$. The T-size equivalents of the conventional CFI cut-off values (poor < 0.90 < fair < 0.95 < close) are poor < 0.868 < fair < 0.928 < close for model: Model 1; T-size RMSEA is computed for $\alpha = 0.05$. The T-size equivalents of the conventional RMSEA cut-off values (close < 0.05 < fair < 0.08 < poor) are close < 0.06 < fair < 0.089 < poor for model: Model 1.).

Inicies and metrics	Value
Comparative Fit Index (CFI)	0.987
T-size CFI	0.979
Tucker-Lewis Index (TLI)	0.983
Bentler-Bonett Non-normed Fit Index (NNFI)	0.983
Bentler-Bonett Normed Fit Index (NFI)	0.980
Parsimony Normed Fit Index (PNFI)	0.757
Bollen's Relative Fit Index (RFI)	0.974
Bollen's Incremental Fit Index (IFI)	0.987
Relative Noncentrality Index (RNI)	0.987
Root mean square error of approximation (RMSEA)	0.052
RMSEA 90% CI lower bound	0.041
RMSEA 90% CI upper bound	0.062
RMSEA p-value	0.378
T-size RMSEA	0.062
Standardized root mean square residual (SRMR)	0.048
Hoelter's critical N (α = .05)	320.161
Hoelter's critical N (α = .01)	360.674
Goodness of fit index (GFI)	0.998
McDonald fit index (MFI)	0.934

Table S2. Standardized factor loadings with confidence intervals (95% CI). (Estimate - estimated factor load; Std. Error - standard error; 95% CI - confidence interval of 95%).

Latent factor	Indicator	Estimate	Std. Error	z-value	p-value	95% CI (Upper)	95% CI (Lower)	All	LV
F1 – reading and writinig	One –minute reading	1.000	0.000			1.000	1.000	0.836	18.365
	Reading of nonsense text	0.786	0.031	25.306	<0.001	0.725	0.846	0.787	14.639
	Two-minute spelling	0.134	0.006	23.380	<0.001	0.123	0.146	0.642	2.502
	One-minute writing	0.157	0.006	24.300	<0.001	0.145	0.170	0.723	2.935
	Stringing beads	0.036	0.002	16.805	<0.001	0.032	0.040	0.889	0.668
F2 – working memory	Number sequence backward	1.000	0.000			1.000	1.000	0.526	0.526
	Verbal fluency	3.661	0.163	22.481	<0.001	3.343	3.981	0.491	1.927
	Semantic fluency	6.287	0.316	19.902	<0.001	5.668	6.906	0.633	3.309
	Vocabulary	1.030	0.068	15.075	<0.001	0.896	1.164	0.353	0.542
	Postural stability	-1.855	0.120	-15.509	<0.001	-2.089	-1.620	-0.33	-0.976
F3 - phonological awerness	Rapid naming	1.000	0.000			1.000	1.000	0.523	9.527
	Phonemic segmentation	-0.202	0.009	-23.793	<0.001	-0.219	-0.186	-0.69	-1.928

Table S3. Factor analysis, parameter estimates and 95% confidence intervals for latent variables (Estimate - estimated factor load; Std. Error - standard error; 95% CI - confidence interval of 95%).

Variable	Estimate	Std. Error	z-value	p	Lower 95% CI	Upper 95% CI	All	LV
F1	347.280	20.117	17.263	< 0.001	307.852	386.707	1.000	1.000
F2	0.277	0.022	12.492	< 0.001	0.233	0.320	1.000	1.000
F3	90.758	9.133	9.938	< 0.001	72.858	108.658	1.000	1.000

Table S4. Factor covariance estimates and 95% confidence intervals for latent variable pairs (Estimate - estimated factor load; Std. Error - standard error; 95% CI - confidence interval of 95%).

Variable	Estimate	Std. Error	z-value	p	Lower 95% CI	Upper 95% CI	All	LV
F1 - F2	8.049	0.390	20.616	< 0.001	7.284	8.814	0.821	0.821
F1 - F3	-157.027	6.413	-24.487	< 0.001	-169.596	-144.458	-0.884	-0.884
F2 - F3	-5.043	0.253	-19.917	< 0.001	-5.539	-4.546	-1.006	-1.006

Table S5. Residual variances and 95% confidence intervals for unstandardized variables (Estimate - estimated factor load; Std. Error - standard error; 95% CI - confidence interval of 95%).

Variable	Estimate	Std. Error	z-value	p	Lower	Upper	All	LV
One –minute reading	149.357	39.439	3.787	< 0.001	72.058	226.656	0.301	149.357
Reading of nonsense text	131.351	26.131	5.027	< 0.001	80.135	182.568	0.38	131.351
Two-minute spelling	8.947	0.791	11.311	< 0.001	7.396	10.497	0.588	8.947
One-minute writing	7.871	1.026	7.67	< 0.001	5.86	9.882	0.477	7.871
Stringing beads	2.5	0.147	17.011	< 0.001	2.212	2.788	0.849	2.5
Number sequence backward	0.723	0			0.723	0.723	0.723	0.723
Verbal fluency	11.72	0.363	32.263	< 0.001	11.008	12.432	0.759	11.72
Semantic fluency	16.398	1.56	10.51	< 0.001	13.34	19.456	0.6	16.398
Vocabulary	2.059	0.117	17.613	< 0.001	1.83	2.288	0.875	2.059
Postural stability	7.641	0.326	23.409	< 0.001	7.001	8.28	0.889	7.641
Rapid naming	240.959	12.888	18.696	< 0.001	215.699	266.219	0.726	240.959
Phonemic segmentation	4.031	0.627	6.425	< 0.001	2.801	5.261	0.52	4.031
